# Characteristics and Determinants of the Presence of Respiratory Symptoms among Sewage Workers in Malaysia

**DOI:** 10.1155/2022/8567594

**Published:** 2022-03-14

**Authors:** Kamarulzaman Muzaini, Siti Munira Yasin, Zaliha Ismail, Ahmad Razali Ishak

**Affiliations:** ^1^Department of Public Health Medicine, Faculty of Medicine, Universiti Teknologi MARA, Sungai Buloh, Selangor, Malaysia; ^2^Centre of Environmental Health and Safety, Faculty of Health Sciences, Universiti Teknologi MARA, Puncak Alam, Selangor, Malaysia

## Abstract

**Background:**

The purpose of this study was to determine the relationships of PM 2.5 and H_2_S exposure with the presence of work-related respiratory symptoms among sewage workers.

**Methods:**

A cross-sectional study was conducted in eleven sewage plants located in the central region of Peninsular Malaysia. One hundred ninety-one sewage workers were assessed using the British Medical Research Council (BMRC) questionnaire. Area air sampling was performed in three different sewage plants to measure the following parameters: physical air quality and concentration of PM 2.5 and H_2_S.

**Result:**

Chronic cough (34.0%) was reported as the most common symptom, followed by chronic phlegm (26.2%), shortness of breath (7.9%), and chest tightness (3.7%). There were five significant determinants of the presence of respiratory symptoms among the sewage workers: shift work (AOR 23.50, 95% CI: 1.90–616.52), working at a sludge treatment facility (STF) (AOR 25.46, 95% CI: 2.06–314.29), a longer duration of working years (AOR 1,21, 95% CI: 1.01–1.44), individual cumulative exposure to PM 2.5 (AOR 9.01, 95% CI: 1.98–83.33), and individual cumulative exposure to H_2_S (AOR 1.04, 95% CI: 1.01–1.07). The majority of STF and non-STF workers had higher exposure to PM 2.5 and H_2_S concentrations in the air than office workers.

**Conclusion:**

Sewage workers working at non-STFs or STFs reported a significantly higher prevalence of work-related respiratory symptoms and exposure to PM 2.5 and H_2_S compared with office workers. Exposure-response relationships were also found in sewage workers' cumulative exposure to PM 2.5 and H_2_S and the presence of respiratory symptoms.

## 1. Introduction

Each county around the world has its own sewerage system managed by sewerage companies, which maintain and ensure the continuity of wastewater treatment. In Malaysia alone, over 50,000 workers are involved in the sewerage and waste management industry. The sewage industry in Malaysia has undergone an impressive revolution in sewage treatment technologies. In the 1950s, primitive or primary sewage treatment in Malaysia used pour-flush and septic tanks. Next, partial or full secondary treatment was introduced during the 1980s to 1990s, namely, the Imhoff tank, oxidation pond, aerated lagoon, and activated sludge or biological filters. In the era of 2000, the technology of a fully mechanized sewage plant was established. With the evolution of the Malaysia sewerage system over the century, the working conditions of the workers in this industry also have changed. Various studies have widely demonstrated that sewage treatment plants (STPs) produce various occupational hazards. These hazards are generated through various sewage plant processes that are implemented to remove contaminants from wastewater or are produced as byproducts [1, 2]. The gases, fumes, mists, and dust generated from various processes, such as mechanical filtering, screening, aeration, and drying the sludge to treat sewage water, are some of the occupational hazards that have adverse effects on sewage workers' respiratory health. Therefore, sewage workers are at high risk of experiencing a broad range of adverse health impacts, primarily occupational lung diseases. They are likely to be exposed to various occupational respiratory hazards ranging from specific chemical agents to microbiological agents [3]. From this review, the authors found that the sewage workers have a higher risk of developing respiratory symptoms and impaired pulmonary function tests (obstructive pattern) especially among those with longer employment. However, none of the selected manuscripts in the review tried to discuss the association between the workers' exposure duration and the hazards concentrations and their effect on their respiratory health. In addition, the emissions of chemicals and pollutants affect not only the respiratory health of workers but also the surrounding environment.

Hydrogen sulfide (H_2_S) and inhalable organic dust such as endotoxin and particulate matter 2.5 (PM 2.5) have been reported to be found in sewage plants and pose a significant risk of negative respiratory health effects among sewage workers. The smaller the size of the dust or particles, the greater the effects on respiratory health [4–6]. These particles will not only contribute to the development of respiratory symptoms but will also cause impairment of lung function if the exposure is prolonged. Reported respiratory symptoms include cough, sore throat, phlegm, runny nose, wheezing, and shortness of breath [7–14]. In addition, to a certain extent, there is also evidence that exposure to these particles can lead to a significant deterioration in sewage workers' lung function, such as a reduction in the predicted FEV1%, predicted FVC%, and FEV1/FVC ratio values [2, 11–13, 15, 16]. Infection, allergic response, inflammatory response, and chemical sensitization along the respiratory tract are possible factors that might contribute to the development of respiratory symptoms and lung function impairment [17]. Apart from that, the dose-related effect of the toxic air pollutants in terms of the frequency and duration of exposure could significantly heighten the individual risk of getting respiratory illnesses [18, 19]. Respiratory illnesses among workers due to exposure to occupational respiratory hazards in the workplace will have significant adverse effects on employers, such as an increased rate of sickness-related absenteeism, hospital admission, and reduced productivity [20–22]. Respiratory infection, asthma, and chronic obstructive lung disease (COPD) are some of the common respiratory illnesses that have been reported to be contracted by sewage workers [23–26].

Generally, STPs in Malaysia use mechanical sewage and sludge treatment processes, consisting of two types of treatment processes, namely, primary treatment and secondary treatment. Initially, sewage water will pass through the primary treatment plant to filter out course material and grit. Subsequently, filtered sewage water will pass through secondary treatment, which will undergo several processes, such as aeration and clarification. The sediment sludge produced during the clarifier process will be further processed in a sludge treatment facility (STF) to produce dry sludge. To date, a combination of both mechanical and biological processes is used to treat sewage [27]. However, some developed countries are adopting the latest technologies in wastewater treatment, such as nanotechnology. The usage of nanotechnology materials has numerous advantages in wastewater treatment processes. They can serve as excellent adsorbents for various types of water contaminants, including physical, chemical, and biological contaminants [28–30]. Hence, different sewage plants adopted within these locations produce different air contaminant concentrations as byproducts.

However, it is important to measure the H_2_S and PM 2.5 air concentrations in sewage plants and assess their exposure among sewage workers. To date, no such study has been conducted among sewage workers. Furthermore, studies of the effects of fine particles and toxic gases in the workplace on workers' respiratory health were conducted on different industrial workers. Therefore, the effect of PM 2.5 and H_2_S, which could arise from sewage plants, on sewage workers' respiratory health is unknown in Malaysia. In addition, previous studies conducted among sewage workers did not assess the relationship between the effects of cumulative occupational respiratory hazard exposure and the presence of respiratory symptoms.

This research will fill the gap in knowledge of the effects of cumulative H_2_S and PM 2.5 exposure on respiratory symptoms among sewage workers. In addition, factors associated with the presence of respiratory symptoms among sewage workers, such as sociodemographic profile, job profile, smoking, and safety practices in the workplace, will also be determined. Thus, this study aimed to determine the characteristics and determinants of the presence of respiratory symptoms among operational sewage workers in Malaysia.

## 2. Materials and Methods

### 2.1. Study Population

A cross-sectional study was conducted from February 2021 to April 2021 among operational sewage workers in the central region of Peninsular Malaysia, which includes Selangor, Putrajaya, and Kuala Lumpur. These regions contain 11 sewage plants. The eleven main sewage plants located in the central region of Peninsular Malaysia were selected. For each plant, the job tasks were further divided into three main groups: (i) office/administration, (ii) non-STF, and (iii) STF. The working sites were divided into three as they involved different sewage processes and job tasks between STFs and non-STFs, which have the potential to generate different levels of risk on sewage workers. Sewage workers in the non-STF are involved in earlier sewage treatment processes, which include mechanical screening of the incoming sewage, aeration processes, and clarification processes. STF workers are involved in the final stage of sewage treatment processes, including mechanical sludge dewatering and sludge cake processes. Universal sampling was applied, and study participants were selected based on the following criteria: male and working for at least one year. No female workers were working as operational sewage workers and thus were excluded from this study. One hundred ninety-one subjects were selected and completed the self-administered questionnaire.

### 2.2. Human Assessment (Respiratory Symptoms)

A total of 191 subjects were interviewed using a modified version of the British Medical Research Council (BMRC) questionnaire [31]. This questionnaire was translated into Malay, pretested, validated, and administered by trained interviewers in a previous published study [32]. The questionnaire comprised six main sections: respiratory symptoms, previous medical history, smoking history, occupational history, level of exposure to hazards, and safety practices at the workplace. The questionnaire was modified as the original version of the MRC questionnaire did not have questions assessing the domain of safety practice compliance at the workplace [31]. Regarding the reliability of the BMRC questionnaire that was done among sewage workers, thirteen relevant items in the questionnaire reported Cronbach's alpha ranging from 0.72 to 0.78. Next, the validity of the relevant items in the questionnaire has been analysed using bivariate correlation analysis, which has shown no significant differences in all items.

Regarding respiratory symptoms, chronic cough was defined as experiencing cough symptoms for at least three days a week for at least three months a year for two consecutive years or more [33]. Chronic phlegm was referred to experiencing phlegm production for at least three days a week for at least three months a year for two consecutive years or more. Chest tightness was referred to as pain or discomfort anywhere along the front of the body between the neck and upper abdomen. However, shortness of breath was referred to as breathlessness on exertion, for example, when hurrying on level ground or walking up a slight incline [33]. Finally, the respiratory symptoms among the sewage workers were further classified into one dependent variable. If the subjects experienced one or more respiratory symptoms out of the four symptoms listed in the questionnaire, they were classified as having respiratory symptoms.

Past respiratory illnesses were defined as any history of respiratory diseases diagnosed and confirmed by medical practitioners. These illnesses included pneumonia, emphysema, bronchitis, chronic bronchitis, pulmonary tuberculosis, asthma, pleurisies, chronic obstructive pulmonary disease (COPD), or any previous chest operation.

Regarding occupational history, the subjects were asked about any previous history of working in dusty or chemical-related occupations for more than two years before joining the company. Other questions included the duration working for the current company, working location (office, STF, or non-STF), job title, duration of exposure to particles or gases, type of job shift, and compliance with safety practices. In regard to the job title, we further classified individuals as blue-collar and white-collar workers. Workers who performed predominantly manual work were classified as blue-collar workers, while white-collar workers were workers who performed predominantly professional, desk, managerial, or administrative work [34].

Next, the following definitions of smoking were used: “noncurrent smokers” were defined as people who do not smoke cigarettes at present, and this also includes subjects who were “former daily smokers” (currently a nonsmoker but had previously smoked daily) and “never daily smoker” (currently a nonsmoker who has never smoked daily but is an occasional smoker); and “current smoker” refers to a person who currently smokes a minimum of one cigarette per day for one month or more [35]. The subjects were then grouped into noncurrent smokers and current smokers using the smoking status variable. Next, a Smokerlyzer was used to measure the carbon monoxide (CO) concentration in the sewage workers' lung. It assisted in providing an immediate assessment of the respondent's smoking status. An exhaled CO of more than 6.5 p.p.m. strongly suggests the subject is a smoker [36].

The levels of exposure to the particles or gases were classified into high or low exposure. Safety practices at the workplace were classified into “some time” or “every time”. Five activities were assessed: wearing personal protective equipment (PPE), not smoking during working hours, washing hands before eating, changing clothes, and taking a bath after working and before going back home. The levels of exposure to the particles or gases were classified into high or low exposure.

### 2.3. Environmental Assessment

Area air sampling was conducted in all eleven sewage management plants selected via universal sampling and located in the central region of Peninsular Malaysia. A portable digital anemometer and a calibrated active device EVM-7 Series were used as the air quality assessment tools. A portable digital anemometer was used to measure the air movement parameter. In addition, the EVM-7 Series air assessment tool was used to measure the other air quality parameters, namely, air temperature, air humidity, carbon dioxide, PM 2.5, and H_2_S [37].

In each plant, air quality assessment tools were located at three main work locations: office, non-STF, and STF. The grab air sampling technique was applied based on guidelines outlined by the Department of Occupational Safety and Health (DOSH), Malaysia [38]. These assessment tools were placed 110 metres above the floor and at least 0.5 metres from the walls. The air sampling measurements were conducted during office hours from 8 am to 5 pm, and the duration of each air sampling procedure was 15 minutes at each location. The air sampling was repeated four times (9 am, 11 pm, 2 pm, and 4 pm) to obtain an average reading for an eight-hour working duration, which represents the average daily exposure for each subject. The air quality parameters recorded in this study were air temperature, air humidity, air movement, carbon dioxide, particulate matter 2.5 (PM 2.5), and hydrogen sulfide (H_2_S).

### 2.4. Statistical Analysis

Data were analysed using Statistical Packages for Social Science (SPSS), version 26.0. Descriptive analysis (proportion) was performed according to the two domain-independent groups of variables, including human and environmental assessments. In addition, the cumulative respirable PM 2.5 and H_2_S exposure for each worker who participated in this study was calculated according to the duration of employment at the current job and air concentration recorded during working hours, which is as follows:

∑ [Total concentration of particle exposure for each worker (mg/m^3^) × Duration of employment (year)].

In accordance with the above equation and occupational history, the cumulative respirable PM 2.5 and H_2_S for each subject could be hypnotically estimated to investigate the exposure-response relationship effects between the individual cumulative exposure of occupational reparatory hazards and presence of respiratory symptoms among the sewage workers. Next, lifetime cigarette consumption was term as “numbers of pack years” and is calculated using the following formula:

∑ [Number of cigarettes smoked per day × Number of years smoked (year)]/20.

The dose-response and health risk quantification among operational sewage workers exposure toward hydrogen sulfide have been quantified via hazard quotient (HQ) calculation. HQ > 1 indicates that adverse effects of the exposure to H_2_S are likely to occur. The HQ is calculated using the following formula:

∑ [(C × EF × ED × inH)/(RfC × W × AT)],where C is the concentration in air mg/m^3^; EF is the exposure frequency (days/year); ED is the frequency duration (years); inH is the inhalation rate = 0.20 m^3^/day for adults; RfC is the inhalation reference dose (mg/kg/day); H_2_S = 2 × 10^−3^ mg/kg/day; W is the body weight (kg); and AT is the average time exposure for noncarcinogens (365 days/year × number of exposure years) [39].

Simple and multivariable logistic regression analyses (forward LR) were used to determine the determinants of respiratory symptoms among the sewage workers. Significant independent variables in the simple logistic regression analysis were included in the multivariable logistic regression analysis. A value of *P* < 0.05 was considered statistically significant with alpha = 0.05 and a power of 90%. Age, smoking, previous history of cardiopulmonary disease, and previous working history in dust-exposure-related companies were selected as potential confounders in the present study.

All the air quality parameters recorded in this study were recorded as continuous numbers. The continuous variables were summarized using mean and standard deviation.

## 3. Results

### 3.1. Background of the Subjects

The human and environmental assessment results of the sewage plants are presented in Table 1. One hundred ninety-one male sewage workers with a mean age of 35.4 years were assessed. Almost half of the workers participating in this study were non-Malaysians from Nepal, Indonesia, Bangladesh, and Pakistan. The mean duration of employment among the sewage workers was 5.4 years. Among them, two-thirds of the workers had previously worked for other companies. There were a small number of workers exposed to dusty and chemical gaseous environments. Most of them were blue-collar workers working in operational sewage sites such as STFs and non-STFs, which directly involved sewage processes. Less than half of the workers did engage in shift work, and shift work was more common among workers who worked in STFs. The majority of participants complied with safety practices and frequently wore a mask during the working period. Additionally, only one-sixth of the sewage workers were current smokers.

### 3.2. Respiratory Symptoms

Respiratory symptoms were grouped into four main categories: chronic cough, chronic phlegm, chest tightness, and shortness of breath. Chronic cough was recorded as the most typical symptom (34.0%) among the sewage workers, followed by chronic phlegm (26.2%), shortness of breath (7.9%), and chest tightness (3.7%). Additionally, 44.5% of the workers experienced at least one or more respiratory symptoms in the past twelve months.

The presence of respiratory symptoms among the workers was analysed by univariate and multivariate analyses (Tables 2 and 3, respectively). In univariate analysis, it was shown that age, married status, previously working for another company, longer duration of working, employment at STFs and non-STFs, involvement in a blue-collar type of job, shift work, exposure to particles or toxic gaseous for more than 4 hours per working day, lack of compliance with safety practices, current smoking, abnormal exhaled CO reading, air movement, concentration of CO_2_, concentration of PM 2.5, concentration of H_2_S, and higher values of individual cumulative H_2_S and PM 2.5 exposure were significantly associated with the presence of respiratory symptoms among the sewage workers (Table 2).

The data were further analysed using multivariate analysis (forward LR method) (Table 3). We found that there were five significant determinants of the presence of respiratory symptoms among sewage workers. The determinants were shift work (AOR 23.50, 95% CI: 1.90–616.52), STF work (AOR AOR 25.46, 95% CI: 2.06–314.29), individual cumulative exposure to PM 2.5 (AOR 9.01, 95% CI: 1.98–83.33), a longer duration of working years (AOR 1.21, 95% CI: 1.01–1.44), and individual cumulative exposure for H_2_S (AOR 1.04, 95% CI: 1.01–1.07). Hence, a predictive model using the estimates of the parameters consisting of five determinant variables was developed. The model was found to be statistically significant at *p* < 0.001 and was considered fit; the Hosmer–Lemeshow test was not significant at 0.169 and *p* > 0.05. The five determinants in the model explained 47.8% (Nagelkerke *R*^2^) of the variance in the presence of respiratory symptoms among sewage workers in the central region of Peninsular Malaysia, and this correctly classified the outcome at a rate of 77.5%. This model had acceptable predictability according to the receiver operator characteristic (ROC) curve, with an area under the curve (AUC) of 84.9% [95% CI: 79.6%–90.2%)].

### 3.3. Environmental Assessment

All 5 air quality measurements in this study were described in mean and standard deviation. The mean concentration of H_2_S and PM 2.5 were (2.437 **±** 3.112) and (0.101 **±** 0.087), respectively. The results also showed that a minority (7.3%) of the workers obtained hazard quotient (HQ) values greater than one, indicating that sewage workers might experience adverse health effects from their exposures to H_2_S in the sewage plants.

Table 4 shows the different work locations and concentrations of PM 2.5 and H_2_S. The results revealed that STF and non-STF workers had greater mean exposure concentrations of PM 2.5 and H_2_S than office workers. In addition, STF workers also recorded a higher mean of carbon dioxide exposure concentrations than non-STF and office workers. STFs recorded more inadequate air movement than non-STFs.

## 4. Discussion

In the present study, sewage workers working in STFs and non-STFs were reported to have a higher prevalence of chronic respiratory symptoms than office workers. The most typical respiratory symptom experienced by one-third of sewage workers was chronic cough, followed by chronic phlegm, shortness of breath, and chest tightness. This finding is in accordance with other study findings [8, 12, 15, 40]. We also found that occupational respiratory hazards, i.e., PM 2.5 and H_2_S, also existed in the sewage plants, which were noted to have higher concentrations at STF and non-STF. The sewage workers who were working at STFs had a 25-fold higher likelihood of experiencing respiratory symptoms than those working in an office. Sewage workers working in STFs are involved in the final sewage treatment process, such as sludge dewatering to produce sludge cakes. This process increases the risk of exposure to fine particles among workers. This finding was in agreement with other study findings, which found that sewage workers handling dry sludge had a higher prevalence of respiratory symptoms than non-dry-sludge workers [12]. This might be due to sludge treatment workers being highly exposed to inhalable dust mostly generated in dry sludge processes [41]. On the other hand, in this study, working in non-STFs was not a significant determinant of respiratory symptoms. A lower concentration of inhalable dust, such as PM 2.5, being generated in non-STFs during sewage processes than in STFs might contribute to this finding. In addition, the sewage treatment processes that occur in non-STFs, such as mechanical filtering, aeration, and clarifier processes, were located outside the buildings. Open-air worksites were also reported to have better air movement and ventilation, as was shown in this study. This might assist in diluting the concentration of PM 2.5, subsequently lessening the exposure among non-STF workers compared with that among STF workers [42].

Next, we found that workers who had one-unit higher individual cumulative exposure to PM 2.5 had a nine times higher risk of respiratory symptoms. This means that workers who have a higher cumulative exposure to hazards will have a higher likelihood of experiencing adverse health effects. Several studies reported that endotoxin and inhalable dust generated from sewage plants caused significant respiratory health problems among sewage workers [9, 12, 13]. In contrast, several studies found that exposure to dust particles did not have a significant effect on the respiratory health of sewage workers [10, 43]. One possible reason explaining this was that the concentration of dust particle exposure among the sewage workers was low. However, none of these studies examined the cumulative effects of PM 2.5 exposure on the development of respiratory symptoms among sewage workers. It is imperative to identify the effects of individual cumulative exposure for every hazard, as this may assist in producing better guidelines for safety practice in the workplace. For example, sewage workers who are doing the tasks that potentially lead to exposure to a higher concentration of occupational dust and who have been working for a longer period in the same job need to conduct respiratory health surveillance for the workers more frequently than a yearly basis, especially for those who are having respiratory symptoms or illnesses such as asthma [44]. In addition, they should be supplied with a more efficient mask, such as an N95, to lessen the health risk from exposure to PM 2.5 and protect sewage workers' respiratory health.

The present study found that most of the H_2_S concentration samples reported at STF and non-STF were in the acceptable range, not exceeding the 10 p.p.m. 8-hour time-weighted average (TWA) limit outlined by OSHA [45]. This study revealed that sewage workers working at both sites were exposed to higher concentrations of H_2_S than office workers. Individual cumulative exposure to H_2_S was identified as a significant determinant of respiratory symptoms among sewage workers. We may conclude that sewage workers with a higher individual cumulative exposure to H_2_S had a significantly higher likelihood of having respiratory symptoms. Previous studies conducted among sewage workers demonstrated that H_2_S arising from sewage plants contributed to the development of respiratory symptoms and reduced lung function parameters [8, 11, 16]. However, these findings contradict the finding of another study in which the authors did not find any significant relationship between H_2_S exposure and the presence of respiratory symptoms [7]. This might be because the authors only determined personal exposure to H_2_S without considering the duration of exposure among the workers. None of these studies examined the effects of individual cumulative exposure to H_2_S on respiratory health. The present study proved that the determination of individual cumulative concentrations of exposure to H_2_S is a vital component that needs to be emphasized to examine its effects on sewage workers' respiratory health. In addition, we calculated the HQ for H_2_S for every worker to determine the health risk effect. The crude analysis showed that sewage workers who had a higher ratio of potential exposure to H_2_S were reported to have a significantly higher prevalence of respiratory symptoms than those who had an acceptable ratio of potential exposure to H_2_S. However, the HQ for H_2_S was not a significant determinant of the presence of respiratory symptoms among sewage workers in the final model.

Next, sewage workers who have longer working years in the sewage industry had a higher likelihood of having respiratory symptoms. This finding indicates that workers' duration of working years is an important determinant for the presence of respiratory symptoms among these workers. One possible reason was that the longer duration of working years would heighten the exposure duration to occupational respiratory hazards. This finding was supported by other research concluding that prolonged exposure to occupational respiratory hazards, such as dust, with a longer working years could heighten the risk of developing respiratory disorders and lung function impairment [46]. According to further analysis, workers working at non-STFs and STFs recorded higher daily exposure to occupational hazards such as PM 2.5 and H_2_S than office workers. Therefore, complying with safety practices in the workplace, for example, wearing suitable personal protective equipment (PPE) such as N95 masks, reduced the risk of exposure to PM 2.5 and H_2_S among the sewage workers even though this was not a significant determinant of the presence of respiratory symptoms in this study. Compliance with safety practices in the workplace has immense effects in protecting workers from unnecessary exposure to hazards in the workplace [47]. In this study, we found that most workers complied with safety practices such as wearing PPE, taking a shower at the end of the working day before they go home, washing hands before eating, and not smoking in the workplace compound. The minority of the workers who failed to comply with safety practices failed to comply with practices related to smoking at work, and some received a safety briefing every morning by their supervisors prior to work commencement.

Sewage workers doing shift work had a 24 times higher risk of having respiratory symptoms than workers in normal shifts. In this study, we found that workers working in STFs were required to work in two shifts: morning shifts and night shifts. This finding is in agreement with other study findings, having found that shift work is related to negative health effects. The risk is related to sleep deprivation and circadian rhythm disruption, which will make the respiratory system less capable of responding to inflammation or infection during the night [48]. Furthermore, night shift workers were shown to have an increased risk of asthma and to be more susceptible to respiratory infections [21, 49]. Another possible reason is that night shift workers usually do not comply with safety practices due to poor monitoring by their superiors compared to workers who work during the morning shift [50]. Nonetheless, evidence in these areas is still scarce.

The findings from this study may provide baseline data regarding the effects of occupational respiratory hazard exposure on the presence of sewage workers' respiratory symptoms. This evidence will assist relevant stakeholders in improving the overall working environment in the sewage industry and ultimately protect sewage workers' respiratory health. Under Section 15 of the Occupational Safety and Health Act 1994 (OSHA) and Section 17 of OSHA, employers' general duties and responsibilities to their employees and persons other than their employees were clearly stipulated. The U.S. Department of Labor Occupational Safety and Health Administration emphasized the importance of employers' or building owners' responsibilities to ensure good indoor air quality [51]. This responsibility contributes to a productive and dynamic environment for building occupants, giving them a sense of comfort, health, and well-being. Hence, to improve the air quality in sewage plants based on the present study findings, the key strategies include reducing the concentration of and duration of exposure to PM 2.5, H_2_S, and other identified hazards. These strategies will significantly reduce workers' cumulative exposure and thus prevent and minimize the adverse effects on sewage workers' respiratory health.

A few recommendations are proposed from this study. The recommendations outlined are based on the “Hierarchy of Control” [52]. First, it is unlikely and not feasible to employ an elimination strategy to remove H_2_S and PM 2.5 from the air by replacing sewage processes with other alternative processes. Thus, reducing the risk by substituting less hazardous methods or processes can be adopted as the best strategy to control hazards in sewage plants. For example, the employer could adapt the usage of nanotechnology in the sewage process and substitute it with the current technology being utilized for sewage processes. Adopting the latest technology, such as nanotechnology, will further reduce the production of potential occupational respiratory hazards from sewage processes. Next, in regard to engineering control, employers should install and ensure proper maintenance of the odour control system (OCS) at work locations with higher probabilities of generating noxious gases. The OCS will assist in adsorbing noxious gases and reduce the air concentration of hazardous contaminants [53]. If the plant site is located in an enclosed building in which all the processes take place, employers should install a suitable exhaust fan to ensure good air ventilation and airflow change. This change may further dilute the harmful gases and inhalable dust found in sewage plants and reduce their concentration in the air [54]. We found that most air movement samples recorded in STFs were poor and probably contributed to exposure to a higher concentration of PM 2.5 and H_2_S among the workers.

Third, the application of administrative controls with engineering control may provide better control of hazards in the workplace. Therefore, a medical surveillance programme should be introduced in the workplace to monitor and protect workers more closely to prevent excessive exposure to these hazards and individuals from working in the same job for a longer period of time than necessary [55]. Pre-employment and periodic medical examinations must be carried out at these sites. The examination will enable employers to identify workers with a higher risk of exposure to hazards, especially those with pre-existing medical diseases related to the cardiopulmonary system. Regarding work shifts, employers should avoid changing the shifts too quickly by introducing less frequent changes in shift rotation for these workers. For example, scheduling shift changes once per month and ensuring that changes do not occur within shorter duration. This initiative will assist workers' bodies in physiologically adapting to changes [56, 57]. Fourth, it is imperative that employers take appropriate initiatives to provide suitable PPE and adequate training to workers on how to use PPE effectively to minimize exposure to noxious agents such as H_2_S and PM 2.5.

This study had some limitations. First, the “healthy workers effect” bias might play a role in masking our study findings. Generally, the working population is healthier than the general population. This may lead to an underestimation of the association between exposure to occupational respiratory hazards and respiratory health effects. Second, only area air sampling methods were employed rather than personal air sampling to measure the air quality parameters and hazardous material concentrations in the air. This was due to budget and time limitations. Future studies should consider using more accurate measurements of cumulative exposure to ensure that the findings are more robust. Next, biological air sampling to measure airborne organisms was not conducted in the present study. Several environmental assessment studies performed in sewage plants showed that sewage workers working in non-STFs had a significantly higher risk of exposure to airborne organisms than those working in STFs [23, 58–61]. Prolonged exposure to airborne organisms may also have harmful effects on an individual's respiratory health. We recommend measuring the biological and chemical contaminants in the environment of the sewage plants simultaneously to assess the effects they have on sewage workers' respiratory health; this shall produce a more reliable model.

Despite the limitations mentioned above, this study has several strengths. The first strength is the sample size. We obtained a large number of participants from eleven of the main sewage plants located in the central region of Peninsular Malaysia. Our study was thus representative of all sewage workers in sewage plants in Peninsular Malaysia. Second, this study attempted to highlight the importance of determining individual cumulative exposure to hazardous materials and its effects on respiratory symptoms. There is a lack of published studies exploring this topic among sewage workers. In addition, this study measured two essential occupational respiratory hazard substances that can usually be found in sewage plants: PM 2.5 and H_2_S. Very few published studies conducted at sewage plants measured both hazardous substances at once and investigated their effects on sewage workers' respiratory health. Therefore, the findings of this study provide important knowledge, especially for those interested in this field.

## 5. Conclusion

In conclusion, sewage workers working at sewage processing sites, both non-STFs and STFs, are exposed to a significant amount of occupational respiratory hazards such as PM 2.5 and H_2_S. These exposures might cause work-related respiratory symptoms among sewage workers. Five determinants were identified to be strongly associated with the presence of respiratory symptoms among the sewage workers: were working at STFs, having a longer duration of working years in the sewage industry, shift type working hours, and higher individual cumulative exposure to PM 2.5 and H_2_S. To protect sewage workers from occupational hazards and prevent the development of work-related respiratory symptoms, employers need to implement changes in the workplaces that can minimize hazard exposure among workers. Finally, employees are also required to take initiative to protect themselves from excessive hazardous exposure by complying with health and safety practices, as outlined by the employers and the occupational safety and health (OSH) team within the organization.

## Figures and Tables

**Table 1:** Descriptive results from human and environmental assessments performed in sewage plants located in the central region of Peninsular Malaysia (N = 191).

Variables	*n* (%) or mean ± SD
*a) Human Assessment*	
Sociodemographics	
Age	35.4 **±** 9.96

Nationality	
Malaysian	98 (51.3)
Non-Malaysian	93 (48.7)

Level of education	
No formal education	14 (7.3)
Primary	28 (14.7)
Secondary	75 (39.3)
Tertiary	74 (38.7)

Marital Status	
Single	59 (30.9)
Married	132 (69.1)

Job Profile	
History of previous work	
No	66 (65.4)
Yes	125 (34.6)

Duration of working (years)	5.4 **±** 5.17
Working site	
Office	41 (21.5)
Non-STF	92 (48.1)
STF	58 (30.4)

Job type	
White collar	36 (18.8)
Blue collar	155 (81.2)

Type of job shift	
Nonshift	113 (59.2)
Shift	78 (40.8)

Exposure to particles per day	
Equal to or less than 4 hours	66 (34.6)
>4 hours	125 (65.4)

Safety practice	
Every time	160 (83.8)
Sometimes	31 (16.2)

Risk and comorbidities	
Hx of previous pulmonary disease	
No	186 (97.3)
Yes	5 (2.7)

Smoking status	
Noncurrent smoker	160 (83.8)
Current smoker	31 (16.2)

Exhaled CO status	
Normal	165 (86.4)
Abnormal	26 (13.6)

Smoking duration (pack years)	27.55 **±** 18.22

*b) Environmental assessment*	
Air humidity	57.253 **±** 0.587
Air movement	0.318 **±** 0.154
Carbon dioxide	1051.094 **±** 372.462
Particulate matter 2.5	0.101 **±** 0.087
Hydrogen sulfide	2.437 **±** 3.112

Hazard quotient	
Acceptable	177 (92.7)
Nonacceptable	14 (7.3)

**Table 2:** Univariate analyses of the presence of respiratory symptoms among sewage workers (N = 191).

Variables	*n* (%) or mean ± SD		Crude OR (95% CI)^a^
	Yes	No	
	*n* = 85	*n* = 106	
*a) Human Assessment*			

Sociodemographics			
Age	37.85 ± 10.17	33.42 ± 9.38	1.05 (1.02–1.08)^*∗*^

Nationality			
Malaysian	42 (42.9)	56 (57.1)	1
Non-Malaysian	43 (46.2)	50 (53.8)	1.15 (0.65–2.03)

Level of education			
No formal education	9 (64.3)	5 (35.7)	1
Primary	14 (50.0)	14 (50.0)	0.56 (0.15–2.08)
Secondary	37 (49.3)	138 (50.7)	0.54 (0.17–1.77)
Tertiary	25 (33.8)	49 (66.2)	0.28 (0.09–0.93)^*∗*^

Marital Status			
Single	18 (30.5)	41 (69.5)	1
Married	67 (50.8)	65 (49.2)	2.35 (1.22–4.50)^*∗*^

Job Profile			
History of previous work			
No	29 (43.9)	37 (56.1)	1
Yes	56 (44.8)	69 (55.2)	1.04 (0.57–1.89)^*∗*^

Duration of working (years)	6.99 ± 5.71	4.06 ± 4.28	1.13 (1.06–1.20)^*∗*^

Working site			
Office	4 (9.8)	37 (90.2)	1
Non-STF	42 (45.7)	50 (54.3)	7.77 (2.56–23.58)^*∗*^
STF	39 (67.2)	19 (32.8)	18.99 (5.91–61.07)^*∗*^

Job type			
White collar	3 (8.3)	33 (91.7)	1
Blue collar	82 (52.9)	73 (47.1)	12.36 (3.64–41.99)^*∗*^

Type of job shift			
Nonshift	54 (37.8)	89 (62.2)	1
Shift	31 (64.6)	17 (35.4)	3.01 (1.52–5.94)^*∗*^

Exposure to particles per day			
Equal to or less than 4 hours	63 (50.4)	62 (49.6)	1
>4 hours	22 (33.3)	44 (66.7)	2.03 (1.09–3.78)^*∗*^

Safety practice			
Every time	57 (35.6)	103 (64.4)	1
Sometimes	28 (90.3)	3 (9.7)	16.87 (4.91–57.92)^*∗*^

Risk and comorbidities			
Hx of previous pulmonary disease			
No	81 (43.5)	105 (56.5)	1
Yes	4 (80.0)	1 (20.0)	5.19 (0.57–47.29)

Smoking status			
Noncurrent smoker	57 (35.6)	103 (64.2)	1
Current smoker	28 (90.3)	3 (9.7)	16.87 (4.91–57.92)^*∗*^

Exhaled CO status			
Normal	61 (37.0)	104 (63.0)	1
Abnormal	24 (92.3)	2 (7.7)	20.46 (4.67–89.58)^*∗*^
Smoking duration (pack years)	28.6 ± 419.28	17.3 ± 11.15	1.05 (0.95–1.16)

*b) Environmental assessment*			
Air humidity	58.641 ± 7.111	56.140 ± 8.700	2.56 (0.40–16.57)
Air movement	0.331 ± 0.157	0.308 ± 0.151	1.04 (1.01–1.08)^*∗*^
Carbon dioxide	1140.729 ± 390.956	979.217 ± 342.102	1.001 (1.00–1.002)^*∗*^
Particulate matter 2.5	0.122 ± 0.092	0.084 ± 0.078	23.35 (5.51–99.09)^*∗*^

Hazard quotient			
Acceptable	75 (42.4)	102 (57.6)	1
Nonacceptable	10 (71.4)	4 (28.6)	3.40 (1.03–11.26)^*∗*^
Hydrogen sulfide exposure	3.653 ± 3.552	1.462 ± 2.297	1.33 (1.17–1.52)^*∗*^
Cumulative H_2_S exposure	36.05 ± 50.04	5.48 ± 11.55	1.063 (1.035–1.092)^*∗*^
Cumulative PM 2.5 exposure	592.33 ± 762.66	192.98 ± 203.29	1.004 (1.002–1.005)^*∗*^

Note: ^a^ based on simple logistic regression; and ^*∗*^significant variables in simple logistic regression with a *p* value of ≤0.05

**Table 3:** Multivariate analyses of the presence of respiratory symptoms among sewage workers (N = 191).

Variables	Adjusted OR (95% CI)^b^	*P* value
Duration of working (years)	1.21 (1.01–1.44)^*∗∗*^	0.037
Working site		
Office	—	
Non-STF	2.95 (0.05–167.99)	0.600
STF	25.46 (2.06–314.29)^*∗∗*^	0.012
Type of job shift		
Nonshift	—	
Shift	23.50 (1.90–616.52)^*∗∗*^	<0.001
Cumulative H_2_S exposure	1.04 (1.01–1.07)^*∗∗*^	0.035
Cumulative PM 2.5 exposure	9.01 (1.98–83.33)^*∗∗*^	0.004

Note: ^b^ based on multivariable logistic regression: forward LR method; and ^*∗∗*^ significant determinants in multivariable logistic regression with a *p* value of ≤0.05. The model fits reasonably well, model assumption is met, and there are no interaction between independent variables and no multicollinearity. Model of the presence of respiratory symptoms among sewage workers: presence of respiratory symptoms = −4.625 + 1.349 (cumulative PM 2.5 exposure) + 2.513 (shift type of job) + 0.025 (cumulative H_2_S exposure) + 0.187 (duration of working (in years)) + 3.147 (working at the STF site) − 1.085 (working at the non-STF site).

**Table 4:** Descriptive results for air quality assessment based on different work locations (N = 191).

Air quality parameters	Work location, *n* (%)		
	Office	Non-STF	STF
	*n* = 41	*n* = 92	*n* = 58
Air humidity	47.973 ± 6.427	60.841 ± 6.345	58.121 ± 6.510
Air movement	0.205 ± 0.059	0.433 ± 0.122	0.217 ± 0.108
Carbon dioxide	813.561 ± 183.771	967.467 ± 312.041	1351.655 ± 373.473
Particulate matter 2.5	0.036 ± 0.018	0.081 ± 0.036	0.178 ± 0.115
Hydrogen sulfide	—	2.652 ± 3.259	3.819 ± 3.008

## Data Availability

The data will be made available from the primary author or corresponding author upon a reasonable request. The data contain indirect identifying characteristics (e.g., age). The data will be made available on request to the primary author (Kamarulzaman Muzaini at kaymuz8@gmail.com).

## Abbreviations

BMRC: British Modified Research Council
COPD: Chronic obstructive pulmonary disease
CO: Carbon monoxide
CO_2:_: Carbon dioxide
DOSH: Department of Occupational Safety and Health
FEV1: Forced expiratory volume in 1 second
FEV%: Forced expiratory volume percent of predicted value
FVC: Forced vital capacity
FVC%: Forced vital capacity percent of predicted value
HQ: Hazard quotient
H_2_S: Hydrogen sulfide
OSHA: Occupational Safety and Health Administration
OCS: Odour control system
PM 2.5: Particulate matter 2.5
p.p.m.: Parts per million
PPE: Personal protective equipment
STF: Sludge treatment facility.

## Supplementary Materials

Supplementary Materials Table S1: final model of the presence of respiratory symptoms among sewage workers. Table S2: the predicted probability of AUC for the model of the presence of respiratory symptoms among sewage workers. Table S3: specificity and sensitivity for the model of the presence of respiratory symptoms among sewage workers. Table S4: collinearity statistics for the model of the presence of respiratory symptoms among sewage workers. Table S5: the Hosmer–Lemeshow test for the model of the presence of respiratory symptoms among sewage workers. Figure S1: ROC curve for the model of the presence of respiratory symptoms among sewage workers.
