# Supplementary material for: Characteristics and Determinants of the Presence of Respiratory Symptoms among Sewage Workers in Malaysia
Source: J Environ Public Health. 2022 Mar 14;2022:8567594. doi: 10.1155/2022/8567594 (PMC8938149; doi:10.1155/2022/8567594)
Supplement: Supplementary Materials — Table S1: final model of the presence of respiratory symptoms among sewage workers. Table S2: the predicted probability of AUC for the model of the presence of respiratory symptoms among sewage workers. Table S3: specificity and sensitivity for the model of the presence of respiratory symptoms among sewage workers. Table S4: collinearity statistics for the model of the presence of respiratory symptoms among sewage workers. Table S5: the Hosmer–Lemeshow test for the model of the presence of respiratory symptoms among sewage workers. Figure S1: ROC curve for the model of the presence of respiratory symptoms among sewage workers. [file 8567594.f1.docx]

|  |  |  |  |  |
| --- | --- | --- | --- | --- |

**Supplementary Materials for**

**Characteristics and Determinants of the Presence of Respiratory Symptoms among Sewage Workers in Malaysia**

**Kamarulzaman Muzaini^1^, Siti Munira Yasin^1^, Zaliha Ismail^1^, Ahmad Razali Ishak^2^, Nurhuda Ismail^1^**

^1^ Department of Public Health Medicine, Faculty of Medicine, Universiti Teknologi MARA, Sungai Buloh, Selangor, Malaysia

**^2^** Centre of Environmental Health and Safety, Faculty of Health Sciences, Universiti Teknologi MARA, Puncak Alam, Selangor, Malaysia

Correspondence should be addressed to Siti Munira Yasin; sitimu.yasin@gmail.com

The supplementary materials are pertaining to the result of the determinants of the presence of respiratory symptoms among sewage workers in Malaysia.

This document includes:

- Tables S1- S5

- Figure S1

**Table S1.** Final model of the presence of respiratory symptoms among sewage workers

Variables in the equation

| Variables | B | SE | Wald (df) | Adjusted OR  (95% CI)^b^ | P-value |
| --- | --- | --- | --- | --- | --- |
|  |  |  |  |  |  |
| Constant | -4.625 | 1.101 | 17.651 (1) | - | <0.001 |
| Duration of working (years) | 0.187 | 0.090 | 4.369 (1) | 1.21 (1.01–1.44)** | 0.037 |
| Working site |  |  |  |  |  |
| Office  Non-STF  STF | -  - 1.085  3.147 | -  1.742  1.045 | -  0.913 (1)  6.374 (1) | ref  2.95 (0.05–167.99)  25.46 (2.06–314.29)** | 0.600  0.012 |
| Type of job shift  Non-shift  Shift  Cumulative H2S exposure | -  2.513  0.025 | -  1.667  0.015 | -  11.268 (1)  5.250 (1) | ref  23.50 (1.90–616.52)**  1.04 (1.01–1.07)** | <0.001  0.035 |
| Cumulative PM 2.5exposure | 1.349 | 1.135 | 5.003 (1) | 9.01 (1.98–83.33)** | 0.028 |

|  |
| --- |

- There are five predictors listed in the final model:

1. Cumulative H_2_S exposure
2. Cumulative PM 2.5 exposure
3. Duration of working (years)
4. Working site / plant
5. Job Shift

- The model equation of the presence of respiratory symptoms among sewage workers

**Z=** -4.625 + 1.349 (cumulative PM 2.5 exposure) + 2.513 (shift type of job) + 0.025 (cumulative H_2_S exposure) + 0.187 (duration of working (in years)) + 3.147 (working at the STF site) - 1.085 (working at the non-STF site)

**Figure S1:** ROC curve for the model of the presence of respiratory symptoms among sewage workers


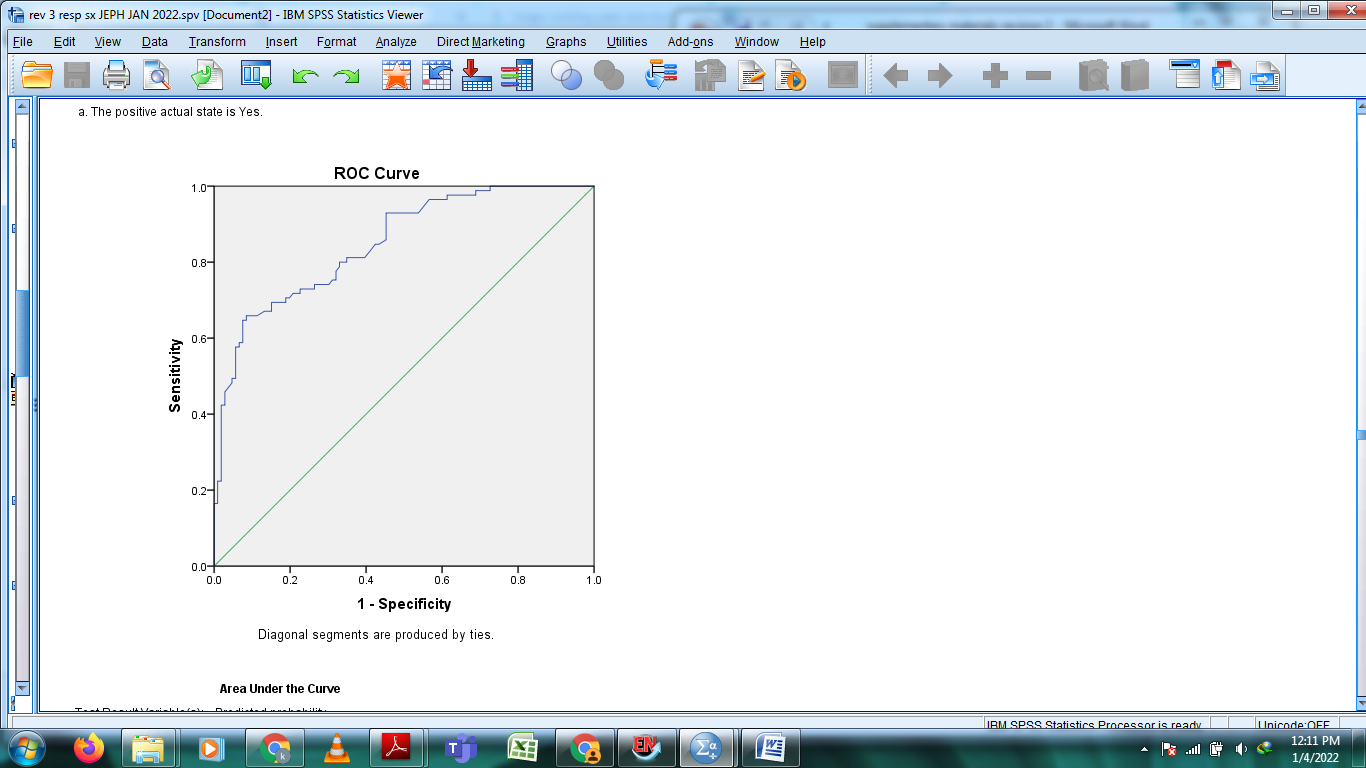


**Table S2.** The predicted probability of AUC for the model of the presence of respiratory symptoms among sewage workers

| **Area Under the Curve** | | | | |
| --- | --- | --- | --- | --- |
| Test Result Variable(s): Predicted probability | | | | |
| Area | Std. Error^a^ | Asymptotic Sig.^b^ | Asymptotic 95% Confidence Interval | |
|  |  |  | Lower Bound | Upper Bound |
| 0.849 | .027 | <0.001 | 0.796 | 0.902 |
| The test result variable(s): Predicted probability has at least one tie between the positive actual state group and the negative actual state group. Statistics may be biased. | | | | |
| ^a^ Under the nonparametric assumption | | | | |
| ^b^ Null hypothesis: true area = 0.5 | | | | |

- The ROC Curve is significant with P-value <0.001, with Area Under the Curve (AUC) for the model of the presence of respiratory symptoms among sewage workers = 84.9% (95 CI%: 79.6 - 90.2).

**Table S3.** Specificity and sensitivity for the model of the presence of respiratory symptoms among sewage workers

| **Classification Table^a^** | | | | | |
| --- | --- | --- | --- | --- | --- |
| Observed | | | Predicted | | |
|  |  |  | presence of respiratory symptoms | | Percentage Correct |
|  |  |  | No | Yes |  |
|  | presence of respiratory symptoms | No | 89 | 17 | 85.8 |
|  |  | Yes | 25 | 60 | 67.1 |
|  | Overall Percentage | |  |  | 77.5 |
| a. The cut value is 0.500 | | | | | |

- From the classification table, the model of the presence of respiratory symptoms among sewage workers has specificity of 85.8% and sensitivity of 67.1% with overall prediction rate of 77.5%

**S4:** Checking and testing for the model of the presence of respiratory symptoms among sewage workers

A) Analysis of interaction between predictors

- There were no significant interaction found between all final predictors variables

B) Analysis of collinearity between predictors

| **Coefficients^a^** | | | | | | | | |
| --- | --- | --- | --- | --- | --- | --- | --- | --- |
| Model | | Unstandardized Coefficients | | Standardized Coefficients | t | Sig. | Collinearity Statistics | |
|  |  | B | Std. Error | Beta |  |  | Tolerance | VIF |
| 1 | (Constant) | -0.326 | 0.126 |  | -2.596 | 0.010 |  |  |
|  | Current working site | 0.201 | 0.044 | 0.320 | 4.596 | <0.001 | 0.755 | 1.325 |
|  | Types of job shift | 0.371 | 0.077 | 0.323 | 4.787 | <0.001 | 0.801 | 1.249 |
|  | Cumulative pm 2.5 mg/m^3^ | 0.070 | 0.068 | 0.080 | 2.031 | 0.034 | 0.611 | 1.636 |
|  | Cumulative H2S exposure | 0.002 | 0.001 | 0.179 | 2.406 | 0.017 | 0.664 | 1.507 |
|  | Duration of working (years) | 0.028 | 0.008 | 0.292 | 3.369 | 0.001 | 0.487 | 2.055 |
|  | | | | | | | | |

**Table S4.** Collinearity statistics for the model of the presence of respiratory symptoms among sewage workers

- Since the variation inflation factor (VIF) for all independent variables are less than 10, Therefore, there were no multicollinearity problem

**S5:** Fit test for the model of the presence of respiratory symptoms among sewage workers

**Table S5.** Hosmer and Lemeshow test for the model of the presence of respiratory symptoms among sewage workers

| **Hosmer and Lemeshow Test** | | | |
| --- | --- | --- | --- |
| Step | Chi-square | df | Sig. |
| 1 | 11.627 | 8 | 0.169 |

- Since the P-value is not significant (p = 0.17), the model the presence of respiratory symptoms among sewage workers

fits well
